# A Rare Heterozygous *TINF2* Deletional Frameshift Mutation in a Chinese Pedigree With a Spectrum of TBDs Phenotypes

**DOI:** 10.3389/fgene.2022.913133

**Published:** 2022-07-07

**Authors:** Hai-Long Ren, Ying-Chun Zheng, Guo-Qian He, Ju Gao, Xia Guo

**Affiliations:** ^1^ Division of Spinal Surgery, Department of Orthopaedics, Nanfang Hospital, Southern Medical University, Guangzhou, China; ^2^ Department of Medical Genetics, School of Basic Medical Sciences, Southern Medical University, Guangzhou, China; ^3^ Department of Pediatrics, West China Second University Hospital, Sichuan University, Chengdu, China; ^4^ NHC Key Laboratory of Chronobiology, Sichuan University, Chengdu, China

**Keywords:** telomere biology disorders, neuroblastoma, TINF2, mutation, phenotypes

## Abstract

Telomere biology disorders (TBDs) induced by *TINF2* mutations manifest clinically with a spectrum of phenotypes, from silent carriers to a set of overlapping conditions. A rare *TINF2* frameshift mutation (c.591delG) encoding a truncated mutant TIN2 protein (p.W198fs) was identified in a 6-years-and-3-month-old Chinese girl with neuroblastoma (NB) by next generation sequencing and confirmed by Sanger sequencing. To explore the possible implications of *TINF2* mutations in TBDs development, the *TINF2* mutant was transfected into the human embryonic kidney (HEK) 293T cells, and mRNA expression of the shelterin complex components as well as the cellular distribution of mutant TIN2 were examined. The *TINF2* mutation was phenotypically associated with short stature in the proband, nail dystrophy and spotted hypopigmentation in her mother, and psoriasis in her older brother. I-TASSER modeling analysis revealed conformational changes of the mutant TIN2 protein and loss of pivotal domains downstream of the 198th amino acid. Additionally, mRNA expression of the shelterin components was downregulated, and TIN2 mutant protein expression was reduced in HEK293T cells transfected with mutant *TINF2*. Furthermore, instead of being restricted to the nucleus, the mutant TIN2 was identified in both the cytoplasm and the nucleus. The *TINF2* gene mutation might impair the function of the shelterin complex and the telomere maintenance mechanisms, both of which are involved in the development of TBDs. TBDs have been associated with increased cancer risk. To the best of our knowledge, this is the first report of NB in patients with TBDs. The relationship between the *TINF2* mutation and NB may need to further study.

## Introduction

Telomere biology disorders (TBDs) are a heterogeneous group of diseases caused by mutations in telomere maintenance genes, with pathogenic germline mutations in at least 15 genes identified to date (Niewish et al., 2022). The TERF1 interacting nuclear factor 2 *(TINF2)* gene (14q12) has been identified as the underlying genetic cause of TBDs with autosomal dominant inheritance. The encoded product TIN2 protein, is a core component of the multi-protein shelterin complex, which protects telomeres (Tomita and Collopy, 2019). TIN2 serves as a hub to bridge various telosome components by directly binding to TRF1 (TERF1), TRF2, and TPP1/POT1 heterodimer. The shelterin complex serves as a platform for recruiting components from several pathways to maintain and protect telomeres (Grill and Nandakumar, 2021).

TBDs caused by *TINF2* mutations clinically present with a spectrum of phenotypes, from silent carriers to a set of overlapping conditions, such as dyskeratosis congenita (DC), Hoyeraal-Hreidarsson syndrome, Revesz syndrome. Incomplete penetrance results in widely varied manifestations, with just one-third of *TINF2*-mutated patients exhibiting typical DC manifestations ^(Cooper and Yong, 2017)^. Moreover, as a haploinsufficient tumor suppressor gene, mutations in *TINF2* and other shelterin genes have been linked to increased cancer risk (Schmutz et al., 2020). Nevetheless, the exact incidence of cancers in patients with *TINF2* mutations is unclear. To date, only a few clinical studies have specifically evaluated the association between cancer risk and specific germline mutations in patients with DC/TBDs and showed that about 1%–4% patients had an underlying *TINF2* mutation (Vulliamy et al., 2011; Schratz et al, 2020; Niewisch et al, 2022).

**FIGURE 1:**
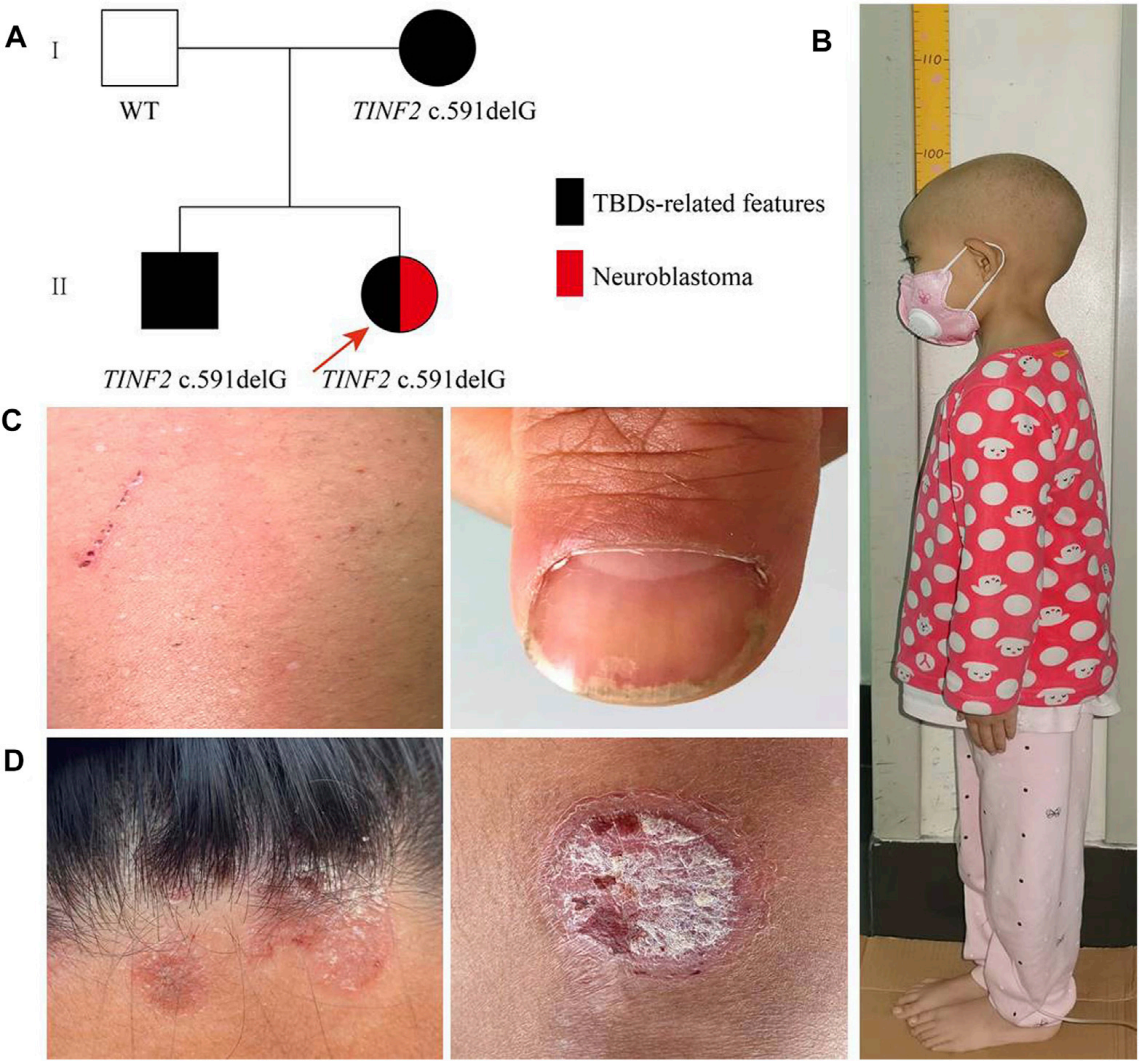
Family pedigree and clinical phenotypes. **(A)** Family pedigree males are marked as squares and females as circles. An arrow indicates the proband. Black symbols, individuals with TBDs-related features; red symbols, individuals with Neuroblastoma. **(B)** The height of the proband was 96.8 cm for 6 years and 3 months old. **(C)** The proband’s mother presented nail dystrophy and depigmentation of the skin. **(D)** The proband’s older brother mainly manifested as red rashes with white scales on the surface on his limbs and hairline, which was aggravated in winter and improved in summer.

Due to limited patient numbers, variable penetrance, and many genetic reasons, determining genotype-phenotype correlation in TBDs is difficult. Here, we reported a Chinese pedigree with a spectrum of TBDs-associated phenotypes (Figure 1A). A rare deletional frameshift mutation resulting in a truncated TIN2-mutant protein (p.W198fs) was documented in the 6-years-and-3-month-old proband with NB, and proven to be pathogenic by *in vitro* functional studies. To the best of our knowledge, this is the first report of NB in patients with TBDs. But, the relationship between the *TINF2* mutation and NB may need to further study.

## Materials and Methods

### Patients

The proband, a 6-years-and-3-month-old girl, was diagnosed with NB and referred to the Department of Pediatric Hematology/Oncology, West China Second University Hospital of Sichuan University for chemotherapy. The clinical data of the patient were collected. The Ethics Committee of West China Second University Hospital has authorized this study. The patient’s family gave written informed consent, and peripheral blood samples were taken from the patient and her family members.

### Candidate Gene Mutation Screening

Genomic DNA was extracted from the peripheral blood of the proband and her family members by a standard phenol/chloroform extraction method. A hematological and neoplastic diseases genes panel was performed on the tumor tissue before chemotherapy from the proband by next generation sequencing (NGS) in collaboration with Kindstar Globalgene Technology (Beijing, China). After the identification of the candidate gene *TINF2*, all family members underwent locus-specific amplification by polymerase chain reaction (PCR) and Sanger sequencing confirmation.

### Bioinformatics

The pathogenic potential of the p. W198fs variant was assessed using Mutation Taster software (http://www.mutationtaster.org/). The I-TASSER service was used to predict the three-dimensional protein structures of wild-type and mutant TIN2 proteins (http://zhanglab.ccmb.med.umich.edu/I-TASSER).

### Construction of Wild-Type and Mutant pEGFP -C1-*TINF2* Expression Vectors

TRIzol (Invitrogen, Carlsbad, CA, United States) was used to extract total RNA from the peripheral blood of healthy family members. First strand cDNA was synthesized using the HiScript II 1st Strand cDNA Synthesis Kit (Vazyme, Jiangsu, China). Primers containing *Eco*RI and *Sma*I (forward primer: 5′-gga​att​ctATG​GCT​ACG​CCC​CTG​GTG​GC-3′, reverse primer: 5′-tccccc​gggTCA​CTC​CTT​TTG​CTC​TGT​GG-3′) sites were designed to amplify the coding sequence of the *TINF2* gene. The PCR products were cloned into the pEGFP-C1 vector (GENEWIZ, New Jersey, United States) through double digestion with *Eco*RI and *Sma*I (New England Biolabs, Beijing, China). The mutant pEGFP-C1-*TINF2*-MUT vector was obtained by PCR-based site-directed mutagenesis using PrimerStar (Takara, Dalian, China). The primers were as follows: forward primer: 5′-GGT​GTG​GAC​ATG​GGT​GGC​TGC​TTC​CAG​AG-3′ and reverse primer: 5′-CTC​TGG​AAG​CAG​CCA​CCC​ATG​TCC​ACA​CC-3’. The constructs were sequenced to confirm that no secondary mutation was introduced. Plasmids were extracted using Endofree Plasmid Midi Kit (Tiangen, Beijing, China).

### RNA Analysis

After reaching 70% confluence in 12-well plates, human embryonic kidney (HEK) 293T cells were transfected with 1 μg of the recombinant plasmid containing the wild-type (pEGFP-C1-*TINF2*-WT) or mutant *TINF2* gene (pEGFP-C1-*TINF2*-MUT) using Lipofectamine™ 2000 transfection reagent (Invitrogen). Thirty-six hours after transfection, total RNA was extracted using the TRIzol reagent (Invitrogen) and reverse transcribed into cDNA using the PrimeScript™ RT reagent kit (Takara). The relative mRNA expression levels of the wild-type and mutant *TINF2* genes were detected by quantitative real-time (qRT) PCR using ChamQ SYBR qPCR Master Mix (Vazyme). *GAPDH* was used as the reference gene to normalize target gene expression. Gene expression levels were calculated by the 2^–ΔΔCT^ method. Transfection and qRT-PCR assays were repeated three times to ensure that the results were reliable. Table 1 presents the primer sequences used for qRT-PCR.

**TABLE 1:** The primer sequences used for the qRT-PCR.

Gene	Forward Primer	Reverse Primer
*TINF2*	CTC​GAA​GCT​GCA​GGA​ACT​TG	CTC​CAG​GCT​GCA​TCC​AAC​TC
*TERF1*	CAG​CTT​GCC​AGT​TGA​GAA​CG	GGG​CTG​ATT​CCA​AGG​GTG​TA
*TERF2*	AAG​CAG​TGG​TCG​AAT​CCA​GT	GTT​GTG​GGG​TCC​TTG​GAC​ATA
*RAP1*	TCC​AGG​AGA​ATG​AAG​AAG​CAG​TC	AAT​CAG​GAG​GGC​TCT​CAT​CCA
*POT1*	GCC​CCC​ATA​TCT​AAG​CAA​AGG​A	TCC​ACT​AAA​GAG​CAG​GCA​AGT
*ACD*	TTG​CAT​CCG​CTG​GGT​GTA​G	CTT​GAG​GTA​CTA​CAG​GAC​GCC
*GAPDH*	GTG​AAG​GTC​GGA​GTC​AAC​G	TGA​GGT​CAA​TGA​AGG​GGT​C

### Western Blot Analysis

After reaching 70% confluence in six-well plates, HEK293T cells were transfected with 2.5 μg of pEGFP-C1-*TINF2*-WT or pEGFP-C1-*TINF2*-MUT using Lipofectamine™ 2000 transfection reagent (Invitrogen). Total proteins were extracted 48 h after transfection using cell lysis buffer (Beyotime Biotechnology, Shanghai, China) and 1% phenylmethanesulfonyl fluoride (Beyotime Biotechnology). The protein concentration was determined using the Pierce™ Rapid Gold BCA Protein Assay Kit (Thermo Fisher Scientific, Massachusetts, United States). Protein loading buffer was added to the cell lysates before boiling for 10 min. Protein samples (30 μg) were then separated by sodium dodecyl sulfate-polyacrylamide gel electrophoresis and used for immunoblotting. The intensities of the protein bands were quantified using ImageJ software (NIH, Maryland, United States), and TIN2 protein expression was adjusted to that of GAPDH (Proteintech, Illinois, United States). Transfection and western blotting analyses were repeated three times for each test.

### Subcellular Localization

After reaching 50%–60% confluence in a confocal dish, HEK293T cells were transfected with 2 μg of pEGFP-C1, pEGFP-C1-*TINF2*-WT, or pEGFP-C1-*TINF2*-MUT using Lipofectamine™ 2000 transfection reagent (Invitrogen). Thirty-six hours after transfection, the cells were washed three times with phosphate-buffered saline, fixed for 30 min with 4% paraformaldehyde (Leagene, Beijing, China), and then incubated in 0.1% Triton X-100 (Thermo Fisher Scientific, Massachusetts, United States) to increase the cytomembrane permeability. Nuclei were stained with 4’, 6-diamidino-2-phenylindole (DAPI; Sigma, St. Louis, MO, United States) to observe the subcellular location of TIN2 proteins. A confocal fluorescence microscope (LSM 880; Carl Zeiss AG, Jena, Germany) was used for cell imaging.

### Telomere Length Analysis

After obtaining informed consent, genomic DNA was extracted from leucocytes from the peripheral blood of the proband and her family members with *TINF2* mutations. The mean telomere length was determined in association with Kindstar Globalgene Technology (Beijing, China) in nearly 30 million telomeres in about 300,000 cells from the samples using Southern blots of terminal restriction fragments (Montpetit et al., 2014). Telomere length is judged according to the percentile in the telomere length database of different age groups: extremely long >80%, long 61%–80%, normal 41%–60%, short 21%–40%, and extremely short <20%.

### Statistical Analyses

Statistical analyses were performed using GraphPad Prism software (GraphPad Software Inc., San Diego, CA, United States). Statistical significance between two groups was determined using the independent samples *t* test. Data are expressed as the mean ± standard deviation (SD). A *p* value <0.05 was considered statistically significant, and the following symbols were used for *p* values: *p < 0.05, **p < 0.01, ***p < 0.001, and ****p < 0.0001.

## Results

### Clinical Report

The proband is 8 years old at the time of writing, and she was delivered after a full-term pregnancy. Her birth weight was 3,100 g, and her birth length was 50 cm. Her parents noticed that her height and weight were lower than those of other children her age and gender when she was 2 years old. She was admitted to the hospital at the age of 6 years and 3 months due to her short stature. A physical examination showed that the proband’s body weight was 10 kg (z-score −3.71, *p* ≤ 0.1); her height was 96.8 cm (z-score −4.55, *p* ≤ 0.1) (Figure 1B); and her body mass index was 10.67 (z-score −2.81, *p* = 0.2). There was no evidence of the classic triad of nail dystrophy, lacy reticular pigmentation of the neck/upper chest, and oral leukoplakia. Routine blood tests revealed no abnormalities. Other regular laboratory tests (including liver and kidney function, blood glucose, pituitary hormones, etc), blood tandem mass spectrometry, urine gas chromatographic-mass spectrometry, karyotype, and bone age determination, and cranial MRI were all negative.

Her father (42-year old) was 167 cm tall; her mother (40-year old) was 160 cm tall; and her older brother (17-year old) was 170 cm tall and weighed 50 kg. The proband’s mother presented only nail dystrophy and depigmentation of the skin (Figure 1C). The proband’s older brother mainly manifested as red rashes with white scales on the surface of his limbs and hairline, which was aggravated in winter and improved in summer (Figure 1D). After a skin biopsy, the proband’s older brother was diagnosed with psoriasis. Both of them showed no hematological changes.

**FIGURE 2:**
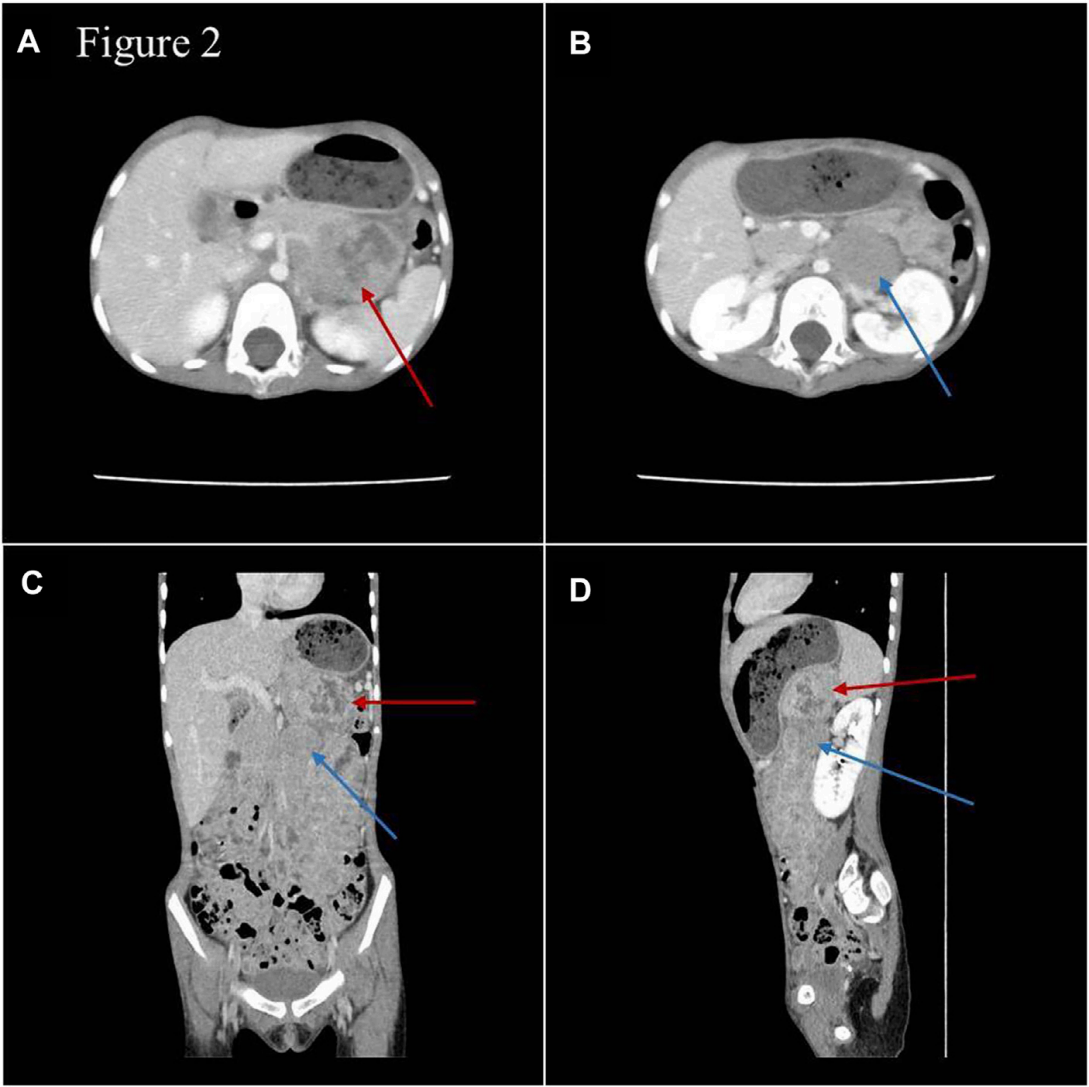
Images of abdominal enhanced CT **(A,B)** and three-dimensional reconstruction **(C,D)**. Space-occupying lesions with inhomogeneous enhancement were in the body and tail of the pancreas (red arrow) and in front of the left renal vein (blue arrow), and the paraaortic lymph nodes were enlarged.

**FIGURE 3:**
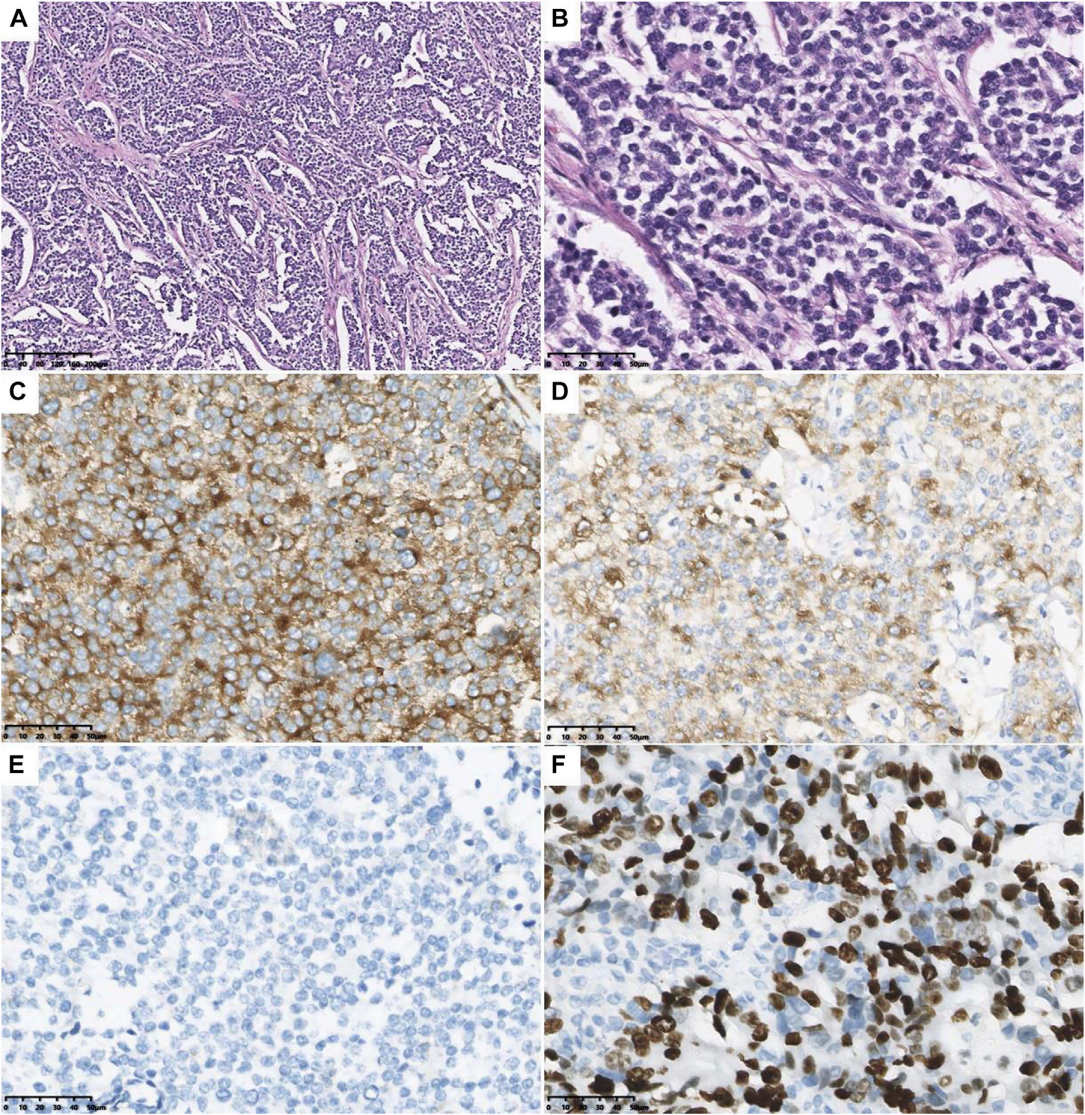
Microscopical and immunohistochemical images of tumor cells. Microscopy revealed the tumor composed of small round blue cells forming rosettes with scant cytoplasm, fibrillary matrix material, and hyperchromatic nuclei **(A)**: H&E staining, original magnification ×10; **(B)** H&E staining, original magnification ×40). The tumor cells are positive for CD57 **(C)** and Syn **(D)**, and negative for CgA **(E)**. The Ki-67 proliferation index was about 25–30% **(F)**.

During hospitalization, an abdominal mass was found because of abdominal pain. Abdominal computed tomography (CT) showed retroperitoneal space occupancy (Figure 2). Pathology confirmed the diagnosis of NB after mass resection. Microscopy revealed a tumor composed of small round blue cells forming rosettes with scant cytoplasm, fibrillary matrix material, and hyperchromatic nuclei (Figures 3A,B). Immunohistochemistry evaluation showed CD57^+^, Syn+, and CgA− tumor cells (Figures 3C–E). The Ki-67 proliferation index was approximately 25%–30% (Figure 3F). Fluorescence *in situ* hybridization showed no *MYCN* amplification. Positron emission tomography/CT was performed postoperatively, which showed no tumor residue. Based on these findings and the onset age, the diagnosis of NB with medium-risk stage III disease was confirmed. Patients with TBDs tend to be more responsive to radiation and chemotherapy treatment than the general population. The side effects of chemotherapy may also be more pronounced. Due to the limited data on the treatment of cancer in TBD patients, there is no guidance on how to carry out chemotherapy according to the diagnosis and management guidelines for DC and TBDs (2015). During the early stages of chemotherapy, the proband experienced no severe hematological toxicity. Therefore, in the follow-up treatment, sufficient radiotherapy and chemotherapy were administered according to the GPOH NB-2017 protocol. At the time of writing, 3 months after treatment completion, the proband is alive and disease-free, and she still has short stature with no manifestation of the classic triad or bone marrow failure.

### Mutation Analysis

**FIGURE 4:**
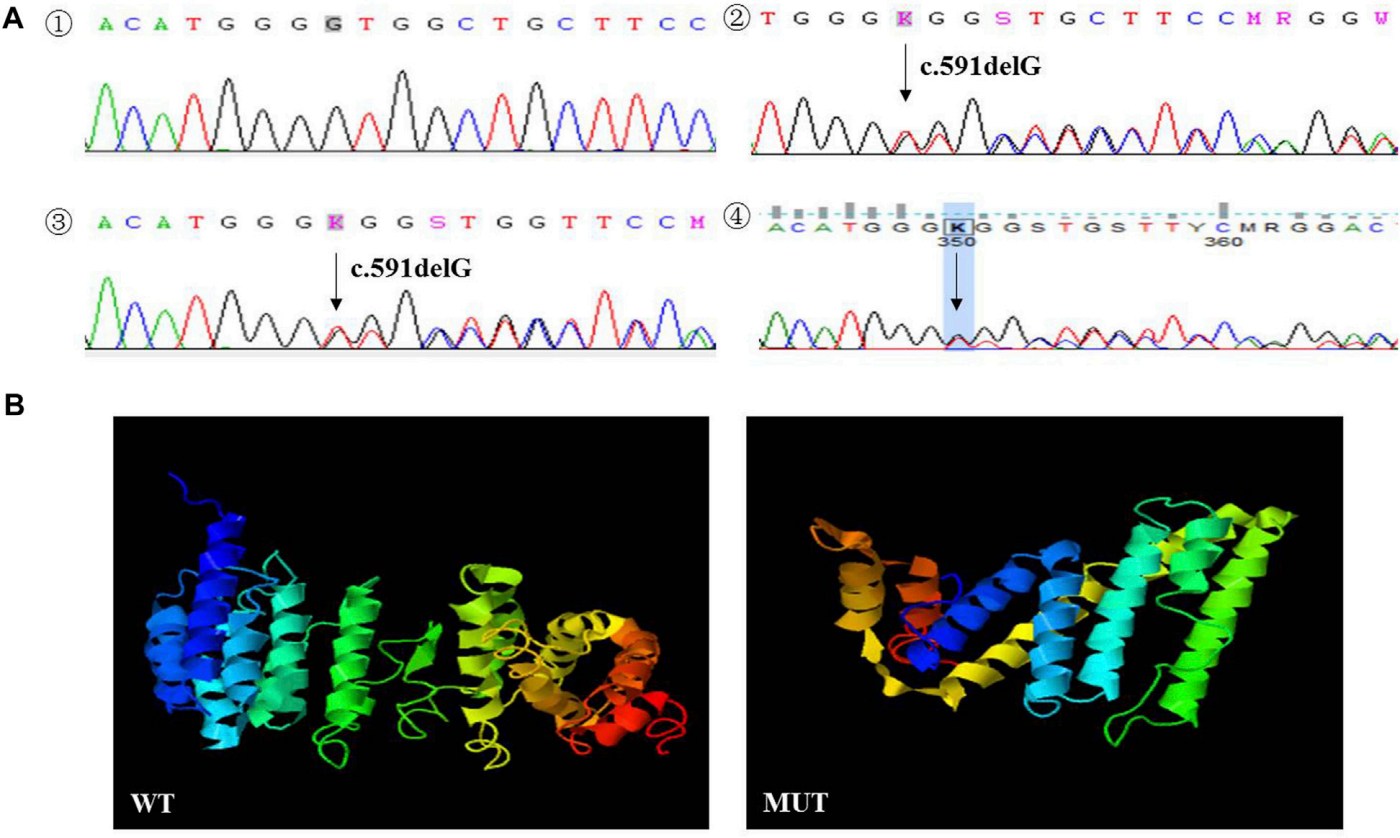
**(A)** Sanger sequencing results of the proband and her family members. The deletion mutation (p. W198fs) in *TINF2* gene was confirmed by Sanger sequencing performed on peripheral blood in the proband (2). The mother (3) and brother (4) of the proband were both carried with this mutation. *TINF2* gene of the father of the proband was wild-type (1). **(B)** Protein structure predicted by I-TASSER: the *TINF2* p. W198fs mutation changed the tertiary structure of the protein.

A rare deletion mutation (c.591delG) of the *TINF2* gene, discovered by NGS on the proband’s tumor tissue before chemotherapy, induced a truncated protein at amino acid position 198. The deletion mutation of the *TINF2* gene was confirmed by Sanger sequencing in the proband and her family members (Figure 4A). No variants were found in other NB common causative genes. The mother and brother of the proband both carried this mutation. Mutation Taster prediction results showed that the base deletion mutant is pathogenic and may interfere with the binding of the TIN2 protein to TERF1 and affect the nuclear localization signal. I-TASSER indicated that the *TINF2* p. W198fs mutation changed the tertiary structure of the protein (Figure 4B). This mutation has not been found in Genome Aggregation Database, the Exome Aggregation Consortium and ClinVar Database at present. According to the ACMG 2015 guidelines (Richard et al., 2015), this mutation is classified as likely pathogenic, supported by PS3, PM2, PM4 and PP3.

### Functional Analysis

**FIGURE 5:**
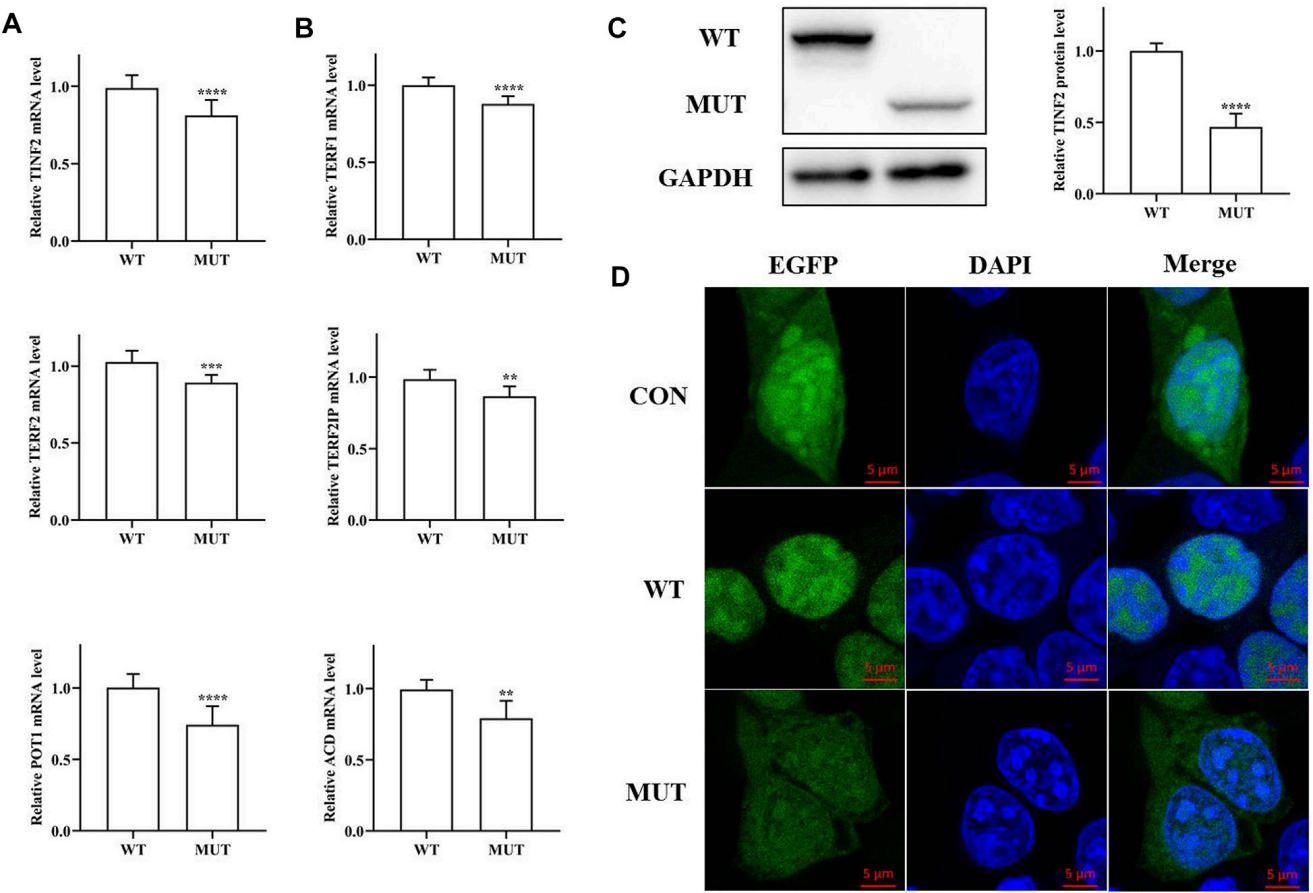
Functional study of the mutated protein. **(A)** mRNA expression level of *TINF2* in HEK293T cells. There is a significant difference in the mRNA level of the wild-type and the mutant (***p < 0.001). **(B)** mRNA expression level of other shelterin genes in HEK293T cells. The expression of other shelterin genes decreased to varying degrees. **(C)**. Protein expression level of *TINF2* in HEK293T cells. The wild-type *TINF2* is expressed at a higher level than the mutant (****p < 0.0001). **(D)**. Subcellular localization of wild-type and mutant *TINF2* in HEK293T cells. Wild-type *TINF2* is localized exclusively to the nucleus. Mutant *TINF2* distribute throughout the cell. Confocal images of EGFP (green), DAPI nuclear staining (blue), and merged signals.

The functional effects of *TINF2* mutations were analyzed in HEK293T cells. Significant differences in mRNA expression levels were observed between cells transfected with pEGFP-C1-*TINF2*-WT and pEGFP-C1-*TINF2*-MUT, as shown in Figures 5A,B (*p* < 0.001). The mRNA expression level of the *TINF2* gene decreased significantly after mutation. Meanwhile, the expression of telomerase protein complex-related genes was also detected. The results showed that after *TINF2* mutation, the mRNA expression levels of other genes (*TERF1*, *TERF2*, *TERF2IP*, *POT1,* and *ACD*) decreased by varying degrees (*p* < 0.01). Western blotting was used to verify the *in vitro* expression of the TIN2 and EGFP fusion protein expression vectors in HEK293T cells (Figure 5C). The expression level of abnormal proteins formed after mutation was significantly lower than that of normal proteins (*p* < 0.0001), and the molecular weight of the mutant protein was significantly lower than that of the wild-type protein.

Differences in the subcellular localization of the mutant and wild-type proteins were also observed (Figure 5D). Green fluorescent protein was expressed in both the nucleus and the cytoplasm; the wild-type TIN2 protein was exclusively localized to the nucleus, whereas the mutant TIN2 fusion protein’s localization was significantly altered; and green fluorescence was observed in both the cytoplasm and the nucleus.

### The Proband DNA Exhibited a Slightly Decreased Mean Telomere Length

**FIGURE 6:**
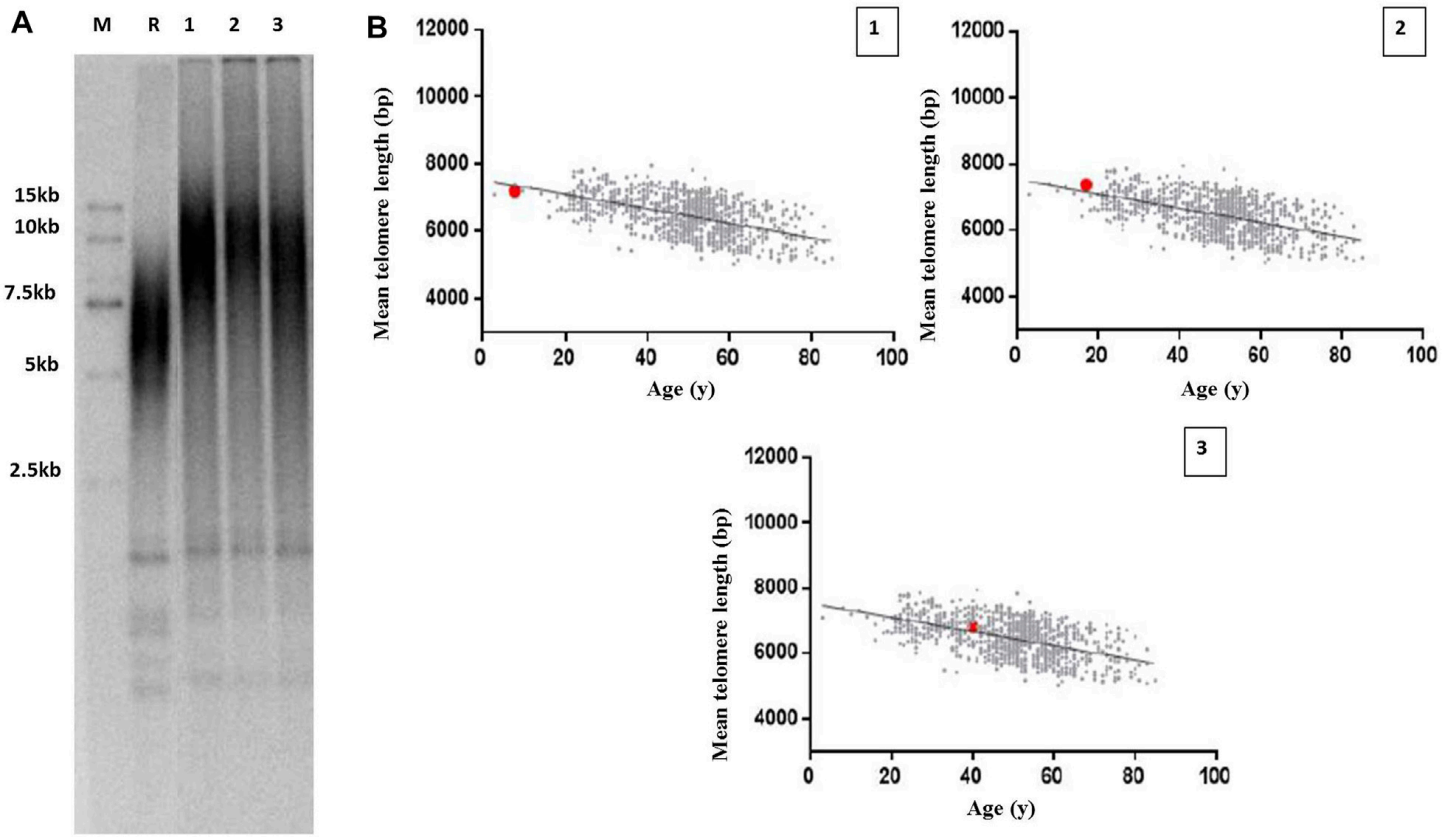
Telomere length measurement in the proband and her family members with *TINF2* mutation. **(A)** Mean leucocyte telomere length was assessed using Southern blots analysis of TRF. M: marker; R: reference standards; 1: the proband; 2: the brother of the proband; 3: the mother of the proband. **(B)** Mean telomere length was quantified according to age in the proband and her family members. The Mean telomere length was respectively 7.2 kb in the proband, the 40th percentile of the same age group (1), 7.38 kb in the brother of the proband, the 58th percentile of the 17-year old population (2), and 6.82 kb in the mother of the proband, the 60th percentile of the 40-year old population (3).

Because of the role of *TINF2* in telomere maintenance, we hypothesized that the mutant *TINF2* could affect telomere length. Therefore, the mean leukocyte telomere length in the proband and her family members with *TINF2* mutations was assessed using Southern blot analysis of terminal restriction fragment lengths. The results showed that the mean telomere length was 7.2 kb in the proband (the 40th percentile of the same age group), 7.38 kb in the older brother of the proband (the 58th percentile of the 17-year old population), and 6.82 kb in the mother of the proband (the 60th percentile of the 40-year old population). When compared with data from the telomere length database of the Chinese population by Kindstar Globalgene Technology, the proband’s mean telomere length was slightly shorter than that of children of the same age, whereas the proband’s mother and brother had normal telomere lengths (Figure 6).

## Discussion

The proband was diagnosed with NB by pathological biopsy because of retroperitoneal space occupancy. A physical examination after admission showed that the proband was short in stature, and her height and weight were below the 10th percentile for children of the same age and sex. There was no evidence of the classic triad of nail dystrophy, lacy reticular segmentation of the neck/upper chest, and oral leukoplakia. Routine examinations, including pituitary hormones and cranial MRI, excluded endocrine-related diseases. Before chemotherapy, NGS was performed to identify molecular genetic abnormalities related to the prognosis. A rare deletion frameshift mutation in the *TINF2* gene, p. W198fs, was identified in the proband. The mother and brother of the proband exhibited skin lesions, while her father had no clinical manifestations. According to Sanger sequencing results, the deletional frameshift mutation was inherited from the proband’s mother, and the proband’s brother also had the same mutation.

Individuals with short telomeres and/or germline mutations in telomere biology genes should be considered to have a TBD (Grill and Nandakumar, 2021; Savage and Bertuch, 2010]. TBD manifestations can range from complicated multisystem disorders that appear in childhood to adults who exhibit one or two DC-related features [Niewisch and Savage, 2019; Kam et al., 2021]. The prevalence of several clinically relevant disease features in patients with *TINF2* mutations has been investigated (Vulliamy et al., 2011). In addition to aplastic anemia (76.7%) and the presence of two or more classical mucocutaneous features (42.9%), short stature is one of the common clinical manifestations of TBDs, accounting for 21.4%. Cancer is more common in people with TBDs; around 1%–4% of patients with *TINF2* mutations have cancer, which tends to affect tissues with rapid turnover (skin, mucus membranes, and bone marrow) (Alter et al., 2009; Vulliamy et al., 2011). NB in patients with *TINF2* mutations has not been reported. Moreover, no psoriasis has been also reported in TBDs patients. Similar to DC, the typical pathological changes of psoriasis are hyperkeratosis/dyskeratosis. A recent clinical study (Mehmet beyoglu et al., 2022) has shown that telomere length in blood cells from psoriasis patients is significantly shorter than that in healthy controls. Telomere shortening may play a role in the pathogenesis of psoriasis. Therefore, we hypothesize that psoriasis is one of the clinical manifestations of TBDs. Functional studies were carried out in this study, which confirmed the pathogenicity of the rare deletional frameshift mutation, p. W198fs, in the *TINF2* gene, due to the atypical clinical features of the proband and her family members. This mutation has been reported previously in patients with thyroid cancer and melanoma (He et al., 2020).

Telomere length abnormalities are increasingly believed to be the underlying cause of clinically detectable disease processes (Stanley and Armanios, 2015). Mutations in the same TBD-related genes, which produce different effects on telomere length, may be the genetic basis of short and long telomere syndromes (Stanley and Armanios, 2015; McNally et al., 2019). The clear connection between short telomeres and degenerative diseases is well known, while new discoveries have uncovered a potential link between long telomeres and familial cancer syndromes (Horn et al., 2013; Robles−Espinoza et al., 2014; Shi et al., 2014). However, several investigations have found that telomeres measured in tumor tissue from the breast, colon, and prostate were shorter than those measured in healthy tissue from the same organs in the same patient (Nakamura et al., 2000; Fordyce et al., 2006; Heaphy et al., 2010). As a result, there is endless controversy over the relationship between telomere length and disease severity (Vulliamy et al., 2011; Vulliamy et al., 2012; Grill and Nandakumar, 2021). Kim et al. (1999) discovered that truncated proteins induced by gene fragment deletion may have varied impacts on telomere length. Unlike deletion of the N-terminal region leading to longer telomeres, C-terminal deletion of amino acids 276–354 slightly shortened the telomere length to approximately 2 kb. In this study, the frameshift mutation also resulted in truncated protein with the C-terminal deletion at amino acids 198. Interestingly, the proband had slightly shorter telomeres, but her family members with the identical mutation had normal length telomeres. Members of this Chinese pedigree also showed different clinical manifestations. The severity of clinical manifestations of the family members seems to be related to their length of telomeres. Variable penetrance and/or expressivity and the presence of genetic anticipation may lead to the clinical and genetic heterogeneity (Vulliamy et al., 2004; Savage et al., 2008). (Vulliamy et al., 2004; Savage et al., 2008).

There is no agreement on the underlying molecular pathogenic mechanisms of *TINF2* mutations in TBDs. All pathogenic *TINF2* mutations in DC patients cluster within a hotspot, among which, those that affect amino acids 280 to 284 are the most common (Savage et al., 2008; Grill and Nandakumar, 2021). The hotspot is located outside of the binding domains and leads to telomere shortening and decreased telomere RNA and telomerase activity, but does not decrease TIN2 binding to shelterin partners (Yang et al., 2011; Frescas and de Lange, 2014). The existence of such a dominant-negative mechanism is supported by experimental data, although the molecular nature of this effect is not fully understood (Grill and Nandakumar, 2021). TIN2 is an essential mediator of TRF1 function, interacting with it via a domain between amino acids 196 and 275. So, the truncated protein may damage TIN2 binding to TRF1 due to loss of binding domains. Additionally, TIN2 is the linchpin of the shelterin complex, which connects TPP1/POT1 to TRF1 and TRF2, and contributes to TRF1 telomere stability. The loss of the TRF1-bingding domain in TIN2 might trigger a DNA damage response (Pan et al., 2021). However, the exact mechanism remains to be further studied.

In conclusion, TBDs are a group of diseases with significant clinical and genetic heterogeneity. Incomplete penetrnce results in highly variable manifestation. The relationship between disease severity and telomere length is still controversial. The underlying molecular pathogenic mechanisms of *TINF2* mutations in TBDs are still not completely understood and remain to be further studied.

## Data Availability

The raw data supporting the conclusion of this article will be made available by the authors, without undue reservation.
